# A Thermosensitive and Degradable Chitin-Based Hydrogel as a Brucellosis Vaccine Adjuvant

**DOI:** 10.3390/polym16192815

**Published:** 2024-10-04

**Authors:** Ruibao Ju, Yanjing Lu, Zhiwen Jiang, Jinhua Chi, Shuo Wang, Wanshun Liu, Yanbo Yin, Baoqin Han

**Affiliations:** 1 Laboratory of Biochemistry and Biomedical Materials, College of Marine Life Sciences, Ocean University of China, Qingdao 266003, China; juruibao@163.com (R.J.); jiangzhiwen@ouc.edu.cn (Z.J.); chijinhua@ouc.edu.cn (J.C.); wangshuo@ouc.edu.cn (S.W.); 13184140693@163.com (W.L.); 2 College of Veterinary Medicine, Qingdao Agricultural University, Qingdao 266109, China; m18809071730@163.com

**Keywords:** hydroxypropyl chitin, temperature-sensitive hydrogel, adjuvant, brucellosis, vaccine

## Abstract

Brucellosis is a zoonotic infectious disease that has long endangered the development of animal husbandry and human health. Currently, vaccination stands as the most efficacious method for preventing and managing brucellosis. Alum, as the most commonly used adjuvant for the brucellosis vaccine, has obvious disadvantages, such as the formation of granulomas and its non-degradability. Therefore, the aims of this study were to prepare an absorbable, injectable, and biocompatible hydroxypropyl chitin (HPCT) thermosensitive hydrogel and to evaluate its immunization efficacy as an adjuvant for Brucella antigens. Specifically, etherification modification of marine natural polysaccharide chitin was carried out to obtain a hydroxypropyl chitin. Rheological studies demonstrated the reversible temperature sensitivity of HPCT hydrogel. Notably, 5 mg/mL of bovine serum albumin can be loaded in HPCT hydrogels and released continuously for more than one week. Furthermore, the L929 cytotoxicity test and in vivo degradation test in rats proved that an HPCT hydrogel had good cytocompatibility and histocompatibility and can be degraded and absorbed in vivo. In mouse functional experiments, as adjuvants for Brucella antigens, an HPCT hydrogel showed better specific antibody expression levels and cytokine (Interleukin-4, Interferon-γ) expression levels than alum. Thus, we believe that HPCT hydrogels hold much promise in the development of adjuvants.

## 1. Introduction

Brucellosis is a global zoonotic infectious disease caused by the invasion of Brucella into the body [1]. In livestock, infection can result in a reduced reproduction and production capacity, subsequently affecting the quality and safety of animal products [2]. People exhibit symptoms such as fever and fatigue following infection [3]. Brucellosis primarily spreads through contact with skin wounds, mucous membranes, and other means [4]. The humoral secretions of infected animals constitute a significant source of brucellosis transmission. In recent years, the incidence of brucellosis has increased rapidly with the increase in livestock feeding. It is estimated that there are more than 500,000 cases of brucellosis worldwide each year. Tetracycline, aminoglycoside, and rifampicin triple combination therapy is efficient in the treatment of human brucellosis [5]. Unfortunately, over the past decades, although researchers have designed many vaccines for human brucellosis, such as live attenuated vaccines, recombinant protein vaccines, vector vaccines and DNA vaccines, the first licensed human brucellosis vaccine is still awaited [6]. The situation of brucellosis prevention and control is very serious. In addition to the mass culling of infected animals, the application of vaccine prevention has become the most effective and economical means of animal brucellosis prevention and control [7].

Vaccines are usually composed of specific antigens and adjuvants. Adjuvants play a critical role not only in carrying antigens but also in augmenting the immune response. The ideal adjuvant should fulfill the necessary physicochemical and biological properties, such as a suitable particle size to facilitate phagocyte uptake, stability to ensure its effectiveness in vivo, autoimmunogenicity to promote an immune response, and biocompatibility to avoid causing excessive toxicity [8]. To enhance the immune response of antigens in the body and to prolong their existence time, adjuvants are usually injected with antigens [9]. At present, the types of vaccine adjuvants include the following: aluminum salt adjuvants, oil emulsion adjuvants, cytokine adjuvants, and some other new immune adjuvants [10]. These current adjuvants widely suffer from problems such as tissue damage, stress, mineral oil residues, toxic reactions, and high costs. The most commonly used adjuvant for brucellosis is alum [11]. Although this adjuvant has the advantages of low cost and convenient transportation [12], its subcutaneous injection facilitates granuloma and abscess formation and it is not easy to be degraded and absorbed [13]. Therefore, it is urgent to develop an ideal adjuvant that can stimulate the body to achieve the strongest immune titer at the smallest dose with high safety, a low price, and few side effects.

Marine natural polymer materials represented by chitin, alginate, and their derivatives have been widely used in the field of medical materials [14]. Studies on vaccine adjuvants have shown that they have large biomolecular loads, easy drug loading, adjustable controlled release capabilities, and unique degradation properties; this makes them promising vaccine delivery systems [15,16,17], in particular, chitin, a naturally bioactive amino polysaccharide. Chitin comes from crustaceans in the ocean and is the second largest biopolymer in nature after cellulose. Chitin can be deacetylated under alkaline conditions to obtain chitosan [18]. Chitin not only has excellent biocompatibility and degradability but is also a typical B cell activator that can promote immune function in humans and animals [19,20].

The ordered structure and various chemical bonds between chitin molecules make it almost insoluble in water and most organic solvents, but the C2-position and C6-position groups in its structure have high activity, which can be chemically modified by carboxymethylation, hydroxylation, and other chemical modifications to form derivatives with various biological activities and good solubility [21]. So far, researchers have explored a lot about chitin and their derivatives as vaccine adjuvants [22,23]. Huajun Zhao et al. found that a chitosan nanovaccine, as a potent carrier-adjuvant system for pIL-12, enhanced HBV-specific CD8^+^ T and CD4^+^ T cell responses and achieved a long-term memory response to HBV, making it a promising candidate for prophylactic HBV vaccine [24]. The application of quaternized chitosan nanoparticles in vaccines is detailed by Shuang Yu et al. [25].

Among these materials, the author believes that temperature-sensitive hydrogels have unique characteristics and advantages as vaccine adjuvants. Their liquid state at low temperatures facilitates antigen encapsulation and injection; when injected into the subject, it can become a solid and stable “reservoir”, which in turn facilitates the sustained release of the antigen. As carriers, temperature-sensitive hydrogels also have excellent effects. For example, Yawen Xu et al. wrapped goat auricular chondrocytes into a hydroxypropyl chitin temperature-sensitive hydrogel to form chondrocyte–hydrogel constructs and injected them subcutaneously into nude mice. The results demonstrated that the HPCH hydrogel possessed satisfactory gelation properties, biocompatibility, and the ability to be applied as an injectable hydrogel for cartilage regeneration [26]. In addition, compared to chitosan, chitin avoids the contamination caused by alkaline deacetylation.

**Scheme 1:**
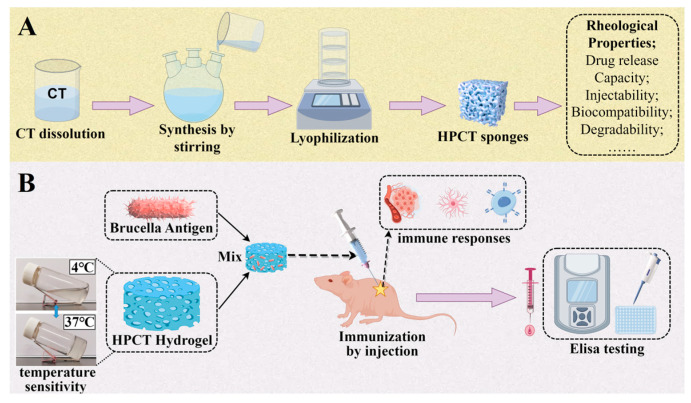
(**A**) HPCT preparation and characterization. (**B**) Hydrogel containing brucellosis antigen for immunization of mice.

Therefore, in this paper, chitin was used as a material and chemically modified to obtain a hydroxypropyl chitin (HPCT) hydrogel with temperature-sensitive properties. The basic physical and chemical properties, rheological properties, drug release capacity, injectability, biocompatibility and degradability were comprehensively evaluated. On this basis, an HPCT hydrogel doped with the Brucella antigen at a concentration of 0.8% was injected subcutaneously into mice, and their serum levels of specific antibody expression and cytokine levels were analyzed by indirect ELISA. Various analyses have shown that HPCT hydrogels have good potential as adjuvants for brucellosis vaccines (Scheme 1). This study provides a preliminary theoretical basis for further exploration of HPCT temperature-sensitive hydrogels as vaccine adjuvants.

## 2. Materials and Methods

### 2.1. Materials and Reagents

Chitin was obtained from Qingdao Biotemed Biomaterial Co., Ltd. (Qingdao, China). 1,2-Propylene oxide was purchased from China Pharmaceutical Group Chemical Reagents Co., Ltd. (Shanghai, China). 3-(4,5-dimethylthiazol-2-yl)-2,5-diphenyl tetrazolium bromide (MTT), pentobarbital sodium, and lysozyme were obtained from Sigma-Aldrich (St. Louis, MO, USA). Dulbecco modified eagle medium (DMEM) was from Gibco^®^, Life Technologies (Carlsbad, CA, USA). Trypsin and fetal bovine serum (FBS) were commercially available from Biological Industries (Kibbutz Beit-Haemek, Israel). Hematoxylin, bovine serum albumin (BSA), and the BCA protein assay kit were commercially available from Solarbio Science and Technology Co., Ltd. (Beijing, China). The mouse interleukin-4 (IL-4) ELISA kit and mouse interferon γ (IFN-γ) ELISA kit were purchased from Shanghai Enzyme-linked Biotechnology Co., Ltd. (Shanghai, China). Alum adjuvant was purchased from Shanghai HuZhen Industry Co., Ltd. (Shanghai, China)

Mouse fibroblast cell lines L929 were supplied by Shanghai Institute for Biological Sciences, Chinese Academy of Sciences (Shanghai, China). Rats and mice, with laboratory animal license number SCXK (Yu) 2019-0002, were purchased from Huaxing Laboratory Animal Farm (Zhengzhou, China). 

### 2.2. Synthesis and Characterization of Hydroxypropyl Chitin

Firstly, the chitin powder was repeatedly frozen and thawed with a sodium hydroxide/urea system to obtain a transparent CT solution. Then, the CT solution was transferred to a three-port flask, 1,2-Epoxypropane was added dropwise at 4 °C and the reaction was stirred for 24 h. The hydrochloric acid solution was added to adjust its pH to neutral, and the purified product hydroxypropyl chitin (HPCT) was obtained by cryodialysis, centrifugation, and freeze-drying. Subsequently, HPCT was validated using the following detection methods.

FT-IR of HPCT was performed on a Nicolet NEXUE470 (Thermo Fisher Scientific Co., Ltd, Waltham, MA, USA) infrared spectrometer using potassium bromide (KBr) tableting method. First, the KBr spectrum was used as the background spectrum. In addition, the HPCT sample and the standard KBr were mixed at a mass ratio of 1:100, and their spectra were measured. The results were analyzed by Omnic software (Version 9.2). 

The one-dimensional ^1^H NMR spectra of HPCT were measured by an Agilent DD2-500 MHz NMR spectrometer (Agilent Technologies (China) Co., Ltd, Beijing, China). HPCT was dissolved in D_2_O at a concentration of 5 mg/mL and transferred to a nuclear magnetic tube for testing. The results were analyzed by MestReNova software (Version 12.0).

The degree of substitution of HPCT is denoted as DS, and the degree of acetylation is denoted as DA. The molecular formulas of HPCT and raw material CT can be expressed as: [(C_6_H_11_NO_4_) (C_2_H_2_O)_DA_ (C_3_H_6_O)_DS_]_n_, [(C_6_H_11_NO_4_) (C_2_H_2_O)_DA_]_n_; Zhang Yongqin optimized the calculation method of substitution degree to the following formula [27]:

$$DA(\%)=\frac{{R^{\prime }}_{C/N}-5.145}{1.715}\times 100\%$$

$$DS(\%)=\frac{R_{C/N}-5.145-1.715\cdot DA}{2.573}\times 100\%$$

${R^{\prime }}_{C/N}$ is the mass ratio of C to N in CT samples, and $R_{C/N}$ is the mass ratio of C to N in HPCT samples.

### 2.3. Analysis of the Properties of HPCT Hydrogel

Different concentrations of HPCT hydrogels were obtained by dissolving HPCT in deionized water and stirring at low temperature.

#### 2.3.1. Thermosensitivity and Injectability Test of HPCT Hydrogels

Different concentrations of HPCT hydrogels (0.5%, 0.8%, and 1.5%) were placed at 4 °C and 37 °C, respectively, and the gelation was observed using the vial inversion method.

In addition, the 0.8% HPCT hydrogel at low temperature was inhaled into a 1 mL syringe equipped with a 26 G (0.45 mm) needle and quickly injected into a Petri dish containing 37 °C warm water to observe the state.

#### 2.3.2. Microstructure Observation of Freeze-Dried Hydrogel by Scanning Electron Microscopy

The 0.8% HPCT hydrogel was quickly frozen in liquid nitrogen after gelation. Then, it was freeze-dried and cut out of the cross-section surface and sprayed with gold, and then it was observed under a scanning electron microscope.

#### 2.3.3. Determination of Rheology Properties of Hydrogels

The rheological properties of HPCT hydrogels were measured with a rotary rheometer (TA Instruments, Waters Technology Shanghai Limited, Shanghai, China), including dynamic strain scanning, dynamic temperature scanning, dynamic frequency scanning, and continuous step strain. All rheology tests were carried out at a gap of 700 μm between a flat plate with a diameter of 40 mm.

Dynamic strain sweep: In order to make sure the rheological measurements were in a linear viscoelastic region (LVR), a dynamic strain scanning from 0.1%~1000% at ω = 1 rad/s and T = 25 °C was carried out prior to other tests. The strain (γ) of 1% was selected to conduct the subsequent oscillation tests. Dynamic temperature scanning from 4 °C to 40 °C at the rate of 2 °C/min with 1% constant strain and 1 rad/s constant frequency was used to estimate the phase transition temperature of hydrogels. 

The temperature sensitivity point of the hydrogel at a concentration of 0.8% was measured to be close to 30 °C by rheometry. The hydrogel at this concentration is liquid at room temperature. Therefore, it is easy to inject and can become a gel after reaching the organism. Therefore, we chose the HPCT hydrogel with a concentration of 0.8% for subsequent related experiments.

### 2.4. Biosafety Evaluation of HPCT Hydrogel

Here, we evaluated the biocompatibility of the HPCT hydrogel in several aspects. This includes evaluation of blood compatibility, cytotoxicity of L929 cells, and in vivo compatibility in rats.

#### 2.4.1. Hemolytic Test

Cytotoxicity studies are important when characterizing novel materials for in vivo biological system interactions. Considering that the materials may be applied in different uses in the future, both 2% red blood cells and whole blood were used to fully evaluate the blood compatibility of this material.

Firstly, red blood cells were isolated from fresh blood of rabbits and diluted with physiological saline to form a 2% red blood cell suspension. For whole blood experiments, the rabbit blood was not washed, and the subsequent operations were the same. The sterilized HPCT was dispersed in a centrifuge tube containing 500 μL sterile saline at a concentration of 50, 500, and 1000 μg/mL. The distilled water was used as a positive control and the normal saline was used as a negative control. A total of 500 μL of erythrocyte suspension or whole blood were added to the samples to be measured and incubated at 37 °C for 1 h. After that, the mixture was centrifuged at 2000 r/min for 5 min, and 100 μL of supernatant was pipetted into a 96-well plate. The absorbance at 405, 530, and 570 nm was measured by a microplate reader [28]. The hemolysis rate (HR) of the material was calculated by the following formula:

$$HR(\%)=\frac{D_{s}-D_{n}}{D_{p}-D_{n}}\times 100\%$$

$D_{s}$ represents the absorbance value of the sample, $D_{n}$ represents the absorbance value of normal saline, and $D_{p}$ represents the absorbance value of distilled water.

#### 2.4.2. Cell Cytotoxicity 

An MTT assay based on mitochondrial function was applied to assess the cytotoxicity of HPCT on mouse fibroblast as reported previously. Briefly, logarithmic growth phase L929 cells were collected and inoculated in 96-pore flat-bottomed plates with 2 × 10^3^ cells/well. After 24 h incubation at the standard conditions, the preliminary media of L929 cells were replaced by a range of concentrations of HPCT (500, 1000, and 2000 μg/mL) for 24 h, 48 h, and 72 h. Cells treated only with complete culture medium were taken as controls. An inverted microscope was used to observe and record cell growth statu. Then, 20 μL MTT solution was added to each well and incubated in an incubator at 37 °C for 4 h. After that, the medium in each well was taken out and 150 μL DMSO was added to each well. The 96-well plate was placed in a microplate reader for incubation and oscillation for 10 min, and the absorbance of each well at 492 nm was measured. The relative cell proliferation rate (RGR) was calculated using the following formula:

$$RGR(\%)=\frac{{OD}_{E}-{OD}_{K}}{{OD}_{C}-{OD}_{K}}\times 100\%$$

Among them, ${OD}_{E}$ represents the absorbance value of the experimental group, ${OD}_{K}$ represents the absorbance value of the blank group, and ${OD}_{C}$ represents the absorbance value of the control group.

#### 2.4.3. Implantation Test in the Subcutaneous Tissues of Rats 

First, a sterile HPCT hydrogel with a mass fraction of 0.8% was prepared. In addition, as the most commonly used brucellosis vaccine adjuvant in the market, alum was used as a positive control material.

Male Sprague Dawley (SD) rats weighing 200 ± 20 g were fed routinely for one week. In order to avoid individual differences, HPCT hydrogel and alum were injected into different areas of the back of each rat. The five observation time nodes of 4 days, 1 week, 2 weeks, 3 weeks, and 4 weeks were set up, respectively, with 5 rats in each node. Rats were anesthetized with pentobarbital sodium, had their back hair shaved before being disinfected with iodophor and alcohol, and fixed on the operating table. Then, 150 μL HPCT hydrogel at 0.8% concentration or alum was injected subcutaneously into the rat’s back using a syringe. After operation, the rats were sacrificed at the designated time point, and the skin containing the material on the back was carefully separated, fixed, and sliced for H&E staining. 

### 2.5. Study on Degradation and Protein Release of HPCT Hydrogel In Vitro

First, we investigated the degradation behavior of HPCT hydrogels by lysozyme solution. Briefly, put 0.8% HPCT hydrogel into the mold to make a cylinder with a diameter of 0.5 cm and a height of 1 cm. The molded gel blocks were placed in a centrifuge tube containing 10 mL lysozyme of different concentrations (100, 200, and 400 U/mL). Enzymatic degradation of the hydrogel was determined at 37 °C and an oscillation speed of 60 rpm. The lysozyme in each tube was replaced once a day. Samples were carefully removed at each fixed time point, freeze-dried and weighed, with three parallel samples in each group. The average value was taken to calculate the degradation rate. The degradation rate (LR) of hydrogel was calculated according to the following formula:

$$LR\left(\%\right)=\frac{W_{0}-W_{t}}{W_{0}}\times 100\%$$

$W_{0}$ is the initial dry weight of the hydrogel, $W_{t}$ is the dry weight of the gel at time t. The results are expressed as mean ± standard deviation.

In addition, the in vitro release of HPCT hydrogels loaded with bovine serum albumin was measured by BCA protein quantification to understand the performance of HPCT hydrogels as carriers. Briefly, HPCT thermal hydrogel with a mass fraction of 0.8% was mixed with 5 mg/mL bovine serum albumin to prepare cylindrical hydrogel blocks of 2 cm in diameter and 2 cm in height. Then, they were put into a sealed conical flask containing 50 mL of physiological saline and shaken in a water bath shaker at 37 °C. At each fixed time point, 100 μL of solution was taken and the same volume of saline was added. The protein content of the solution was determined by BCA protein assay kit and the protein release rate from HPCT hydrogel was calculated. The cumulative released (DR) of BSA was calculated by the following equation:

$$DR\left(\%\right)=\frac{W_{t}}{W_{0}}\times 100\%$$

$W_{t}$ is the mass of BSA released at time t, $W_{0}$ is the total mass of BSA in the gel.

### 2.6. Animals and Vaccinations

The Brucella recombinant protein antigen used in this experiment was provided by Professor Yin Yanbo from Qingdao Agricultural University. This protein antigen is designed by four outer membrane proteins, Omps 16, Omps 19, Omps 25, and Omps 31 [29,30,31]. It is especially pointed out that this protein antigen has been removed from bacteria and endotoxins and does not cause any infections. 

Mice, the classic model for immunological studies, have an immune system that shares many similarities with humans, and they are easy to raise and manipulate. Moreover, there is a relative abundance of immunohistochemical reagents, immunological tools, and reagents for mice, so we selected mice as the donor for this experiment. Female mice (6–8 weeks old, 8 mice per group) were divided into the HPCT hydrogel experimental group (with antigen), commercial alum group (with antigen), PBS group (with antigen), and saline group (without antigen, as a “control group”). The adjuvant–antigen mixture was configured as 40 μg of protein antigen per 100 μL of adjuvant. Each mouse was subcutaneously injected with 300 uL of the corresponding adjuvant–antigen mixture or saline on the back, and at days 14 and 28 after the initial immunization, the same dose and route were used for booster immunization for a total of 3 times. The time point of primary immunization was recorded as 0 week. Blood was taken from the orbit of mice at 0, 2, 4, 6, and 8 weeks, and serum was extracted for later use. 

### 2.7. Determination of Specific Antibody Responses

In this experiment, indirect ELISA was used to evaluate the level of specific antibodies caused by each group of materials. The specific steps are shown in the Supplementary Data. 

### 2.8. Determination of Cytokines in Serum

ELISA kits were used to detect the cytokines IL-4 and IFN-γ in the serum of mice, which were related to the level of immunity, to assess the immunization effect of the hydrogel adjuvant [32,33]. Combined with specific antibody expression levels, the effectiveness of HPCT hydrogels as adjuvants was comprehensively evaluated.

### 2.9. Statistical Analyses 

Data were presented as the means ± standard deviations. Statistical significance was determined by using an independent sample *t*-test. Significant differences between the groups were expressed as follows: * *p* < 0.05, ** *p* < 0.01, and *** *p* < 0.001. The statistical significance was carried out using SPSS version 18 (SPSS Inc., Chicago, IL, USA). 

## 3. Results and Discussion

### 3.1. Hydroxypropyl Chitin Was Successfully Synthesized

**Figure 1:**
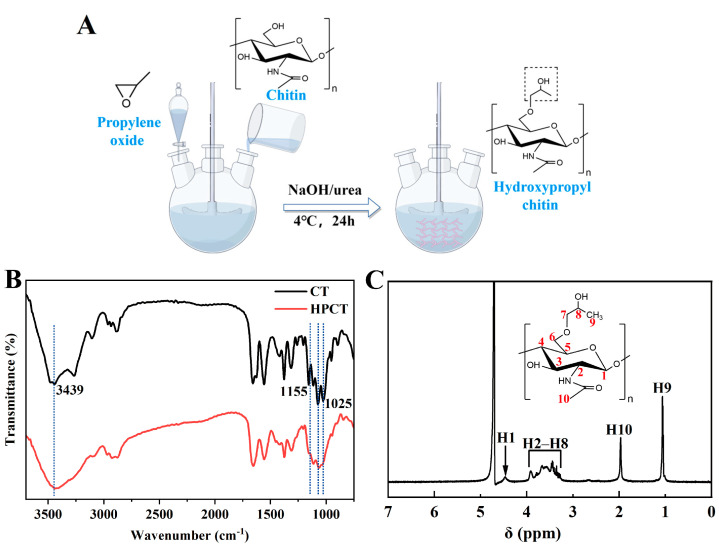
The synthesis route (**A**), FT-IR spectra (**B**), and ^1^H NMR spectra (**C**) of HPCT.

HPCT was prepared according to the chemical reaction route shown in Figure 1A. After chitin was alkalized at low temperature, some hydrogen bonds were destroyed and the active sites were exposed [34], which was beneficial to the synthesis reaction. Subsequently, chitin was etherified and introduced into hydroxypropyl group to obtain hydroxypropyl chitin. Low temperature conditions were also used to prevent deacetylation of chitin. The prepared product had good water solubility.

FT-IR and ^1^H NMR data analysis was used to identify whether hydroxypropyl was grafted on the molecular chain of chitin. The FTIR spectra of chitin and HPCT were depicted in Figure 1B. It can be seen from the diagram that the absorption peaks of primary and secondary alcohols at 1025 cm^−1^ and 1072 cm^−1^ are weakened to varying degrees after the introduction of hydroxypropyl. The absorption peak of HPCT at 3439 cm^−1^ was significantly enhanced, which was the absorption peak of -OH in hydroxypropyl. The absorption peak of the stretching vibration of C-O at the C6 position of chitin was at 1155 cm^−1^, which was significantly weakened in HPCT, indicating that hydroxypropyl group was grafted at the C6 position of chitin [35]. With regard to the ^1^H NMR shown in Figure 1C, the absorption peak at 1.06 ppm (H9) was generated by the hydrogen proton from the methyl group on the hydroxypropyl group and the absorption peak at 1.97 ppm (H10) is attributed to the methyl hydrogen proton on the acetyl group [36,37]. There was no significant difference between the characteristic absorption peaks of CT and HPCT at other positions. These prove that we have successfully synthesized HPCT.

The degree of substitution of hydroxypropyl is a key factor affecting the solubility, gelation, and degradation properties of HPCT. At present, the determination methods of hydroxypropyl substitution degree in the literature mainly include nuclear magnetic resonance [37] and elemental analysis [38]. Chitin is insoluble in most organic solvents and the proton peaks useful for structural analysis of HPCT are susceptible to interference by solvent peaks in the ^1^H NMR method. Therefore, ^1^H NMR was only used to determine the grafting of hydroxypropyl, and elemental analysis was used to further determine the degree of hydroxypropyl substitution.

In this experiment, the elemental analysis results of CT and HPCT by elemental analyzer are shown in Table 1. The degree of acetylation of CT was 96.10%, and the degree of substitution of hydroxypropyl in HPCT was 78.51%, by computing.

### 3.2. HPCT Hydrogel Has Temperature Sensitivity and a Porous Structure

**Figure 2:**
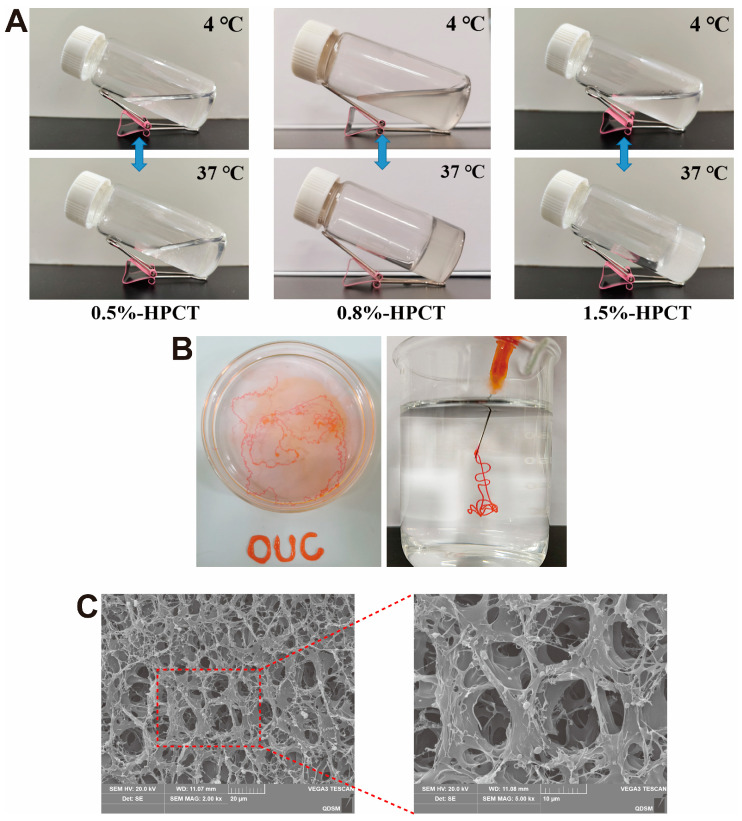
(**A**) Phase transition of HPCT hydrogels with different concentrations under vial tilting method. (**B**) Plasticity and injectability of HPCT hydrogel (0.8%). (**C**) Structural morphology of HPCT hydrogel under scanning electron microscope.

Figure 2A provides visual evidence that HPCT hydrogels are temperature sensitive. It can be seen that HPCT hydrogels have good fluidity at 4 °C. When the temperature rises to 37 °C, HPCT hydrogel solutions at concentrations of 0.8% and 1.5% turn into gels that no longer flow after being placed obliquely in vials. When transferred to 4 °C again, the HPCT hydrogels are transformed into a transparent liquid, the process is reversible, and the hydrogel remains temperature-sensitive after being placed for one month. HPCT hydrogel at a low concentration of 0.5% is semi-gelatinous at 37 °C. Therefore, HPCT hydrogels have good reversible gelation properties. In addition, Figure 2B confirmed that the HPCT hydrogel at a concentration of 0.8% could be injected into 37 °C water through a 1 mL syringe without clogging the needle, and it gelled slowly, suggesting that it has good injectability. Moreover, highly concentrated hydrogels can even be molded into various shapes at room temperature, which proves their plasticity.

The microstructure of HPCT hydrogel after freeze-drying is shown in Figure 2C. It can be seen that the HPCT hydrogel exhibits a uniform and fluffy porous structure, which is conducive to the loading of small molecules and the release of substances. In addition, the homogeneous pores are very favorable for the adhesion and penetration of cells and their interaction with antigens [18].

### 3.3. Rheology Behavior of HTCT Hydrogel

**Figure 3:**
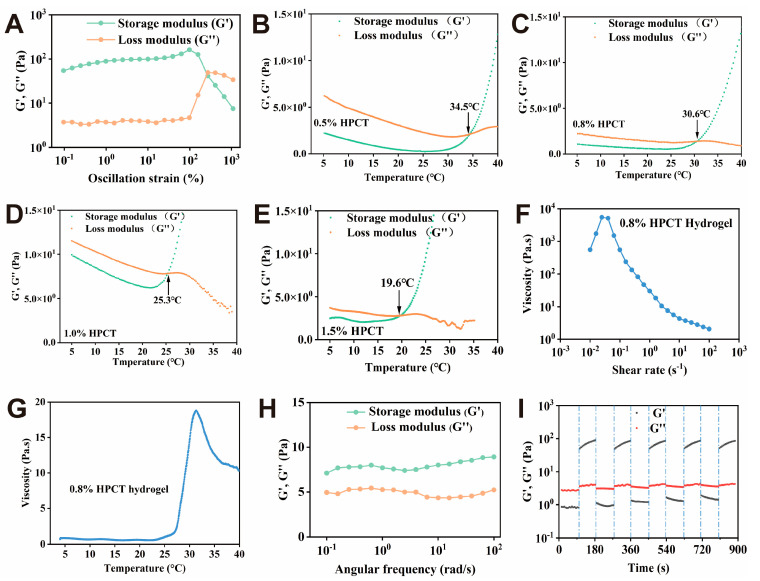
Rheological Behavior of HPCT. (**A**) Dynamic strain scanning of HPCT to determine the linear viscoelastic zone region (LVR). (**B**–**E**) Temperature dependence of storage modulus (G′) and loss modulus (G″) of HPCT hydrogels with different concentrations. (**F**) The correlation between viscosity and shear rate of HPCT hydrogel at 37 °C. (**G**) Viscosity changes in HPCT hydrogel at different temperatures. (**H**) Correlation between dynamic frequency (ω) and modulus of HPCT hydrogels at 37 °C. (**I**) The reversible temperature phase transition ability of HPCT.

First, we carried out dynamic strain scanning of HPCT hydrogel (1%) in the range of 0.1%~1000% to determine the linear viscoelastic region (LVR) of the hydrogel. It was observed from Figure 3A that the hydrogel was stable within the range of 0.1% and 100% strain. To ensure that the measured data were meaningful, we chose a strain of 1% for subsequent testing.

The phase transition temperature of hydrogels with different concentrations were accurately measured by dynamic temperature scanning of a rotary rheometer. Usually this temperature at which the phase transition occurs is referred to as the lower critical solution temperature (LCST) [39]. It is shown as the intersection of storage modulus (G′) and loss modulus (G″) in the image. The phase transition temperature of HPCT with a concentration of 0.5%, 0.8%, 1%, and 1.5% decreased gradually, which were 34.5 °C, 30.6 °C, 25.3 °C, and 19.6 °C, respectively (Figure 3B–E). Considering the temperature sensitivity and operability in complex environments, we selected the HPCT hydrogel with a concentration of 0.8 % for subsequent characterization and animal experiments. The loss modulus of the HPCT hydrogel was greater than the storage modulus at lower temperatures, so the hydrogel behaved as a liquid. As the temperature increases, the storage modulus of the hydrogel gradually exceeded the loss modulus, so it showed in a gel state. This property is speculated to be caused by the entanglement and hydrogen bonding of hydroxypropyl chitin molecular chains at high temperatures [40]. In addition, the modulus of the hydrogel was less than 20 Pa, indicating that its strength was not suitable as a supporting material, but it was feasible for use as a release carrier for substances.

The viscosity of the hydrogel at different shear rates was observed by increasing the shear rate from 0.01 to 100 (s^−1^). The results were shown in Figure 3F. With the increase in shear rate, the apparent viscosity of the sample increased first and then decreased gradually. The viscosity of hydrogel increases at low shear rates. This is due to the fact that under resting or low shear rate conditions (less than 0.1 shear rate s^−1^), polymer chain molecules become entangled with each other and form large intermolecular forces, and when the energy of the physical bonds is similar to the magnitude of the thermal energy, the interactions lead to shear thickening [41]. Alternatively, this could also be a result of the unsteady state shear rate. After all, we started this experiment at a very low shear rate of 0.01. As the shear rate increases further, the viscosity of the hydrogel starts to decrease continuously. It shows that the hydrogel has obvious shear thinning characteristics, so it has appropriate injectability. The viscosity of the hydrogel was measured at different temperatures. It can be seen from Figure 3G that the viscosity of the hydrogel suddenly increased near the temperature sensitive point. After being injected into the body, the high viscosity of the hydrogel and the formed gel state can make the material encapsulated in it stable. 

In order to evaluate the mechanical strength (G′, G″) and frequency dependence (ω) of the hydrogel, we performed dynamic angular frequency scanning of the hydrogel in the range of 0.1–100 rad/s at 37 °C. As shown in Figure 3H, at the angular frequency of 0.1–100 rad/s, the storage modulus (G′) of the hydrogel was greater than the loss modulus (G″), indicating that the HPCT is in a gel state. Under the angular frequency scanning of 0.1–100 rad/s, the modulus of all hydrogels remained relatively stable and did not show a downward trend, indicating that the modulus of hydrogels did not depend much on the frequency. The hydrogel can still maintain a stable gel state at a higher frequency, indicating that the hydrogel is not easy to be destroyed when subjected to external forces.

Moreover, we further demonstrate the excellent temperature-reversible phase transition capability of HPCT hydrogel by Figure 3I. Multiple heating-cooling cycles (4 °C-37 °C-4 °C-37 °C) were performed on the hydrogel, and we monitored the modulus change in the hydrogel at 4 °C and 37 °C. At 4 °C, the HPCT was in the sol state, and at 37 °C, the HPCT was transformed to the gel state. After several cycles, the HPCT hydrogel still had stable temperature sensitivity and modulus data. Hydrogel’s excellent rheological properties allow it to remain stable in complex body environments. 

Adjuvants are used in a variety of ways, but they are most often used by injection. As an injection, adjuvants should meet suitable rheological properties, such as viscosity for tissue adhesion, shear-thinning properties for injection, and stability, etc. In addition to these characteristics, the outstanding temperature sensitivity of the HPCT hydrogel allows for sustained release of the antigen after gel formation. This is not a necessary property for an adjuvant, but this excellent responsiveness deserves to be boasted. We believe that this superior property is perfect as a carrier for the release of substances.

### 3.4. Safety of HTCT Hydrogel

As clinical medical biomaterials, safety is of paramount importance. Only if they are not harmful to the body (good hemocompatibility, low cytotoxicity, appropriate biodegradability) can they be allowed to be used [42,43,44]. Therefore, comprehensive biosafety evaluation of biomaterials is particularly important.

#### 3.4.1. Good Biocompatibility of HPCT Hydrogel In Vitro

The hemolysis test is an important indicator of blood compatibility. Hemolysis is the phenomenon of rupture and dissolution of red blood cells. Materials with good blood compatibility will not cause hemolysis when in direct contact with blood. All injections and pharmaceutical preparations that may cause hemolysis should be subjected to a hemolysis experiment [45,46]. For drugs intended for intravenous administration or materials in direct contact with blood, the use of whole blood to assess the biocompatibility of the material is physiologically more relevant [28].

**Figure 4:**
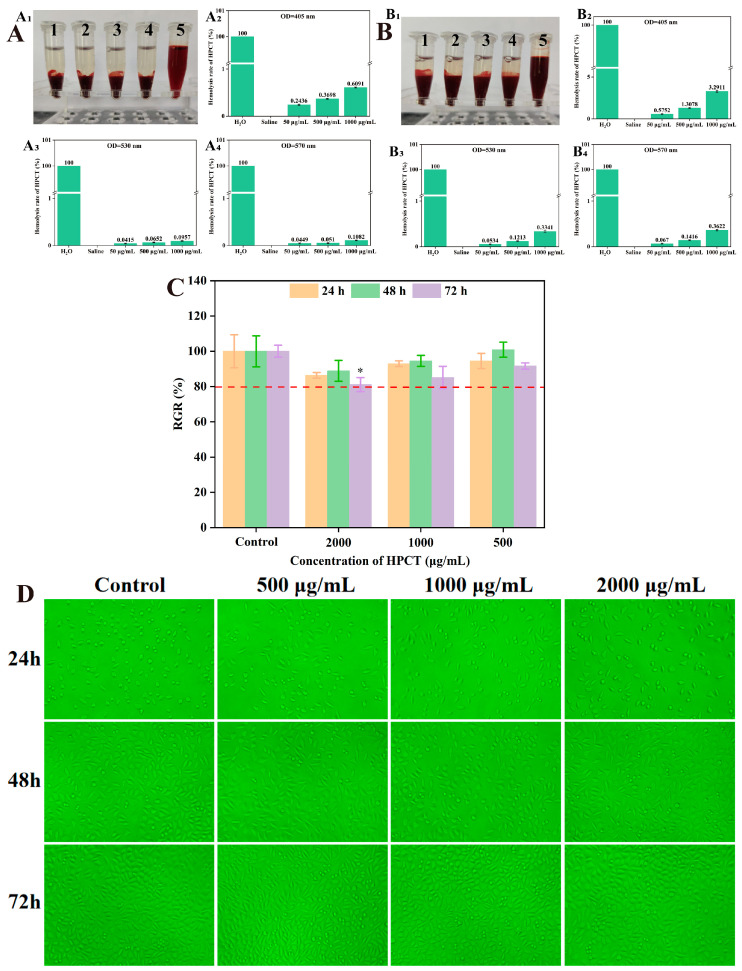
Hemolysis activity and hemolysis assay images of HPCT at different concentrations with pure water as a positive control and saline as a negative control group: (**A**) Tests using whole blood. (**B**) Tests using 2% red blood cells (Numbers 1–5 in (**A**,**B**) represent 50 μ/mL, 500 μg/mL, and 1000 μg/mL of hydrogel, saline, and pure water, respectively). Data represent the mean ± SD (*n* = 5). Effect of HPCT solution on the morphology and proliferation of L929 cells: (**C**) MTT test measured the proliferation rate. (**D**) Morphological changes of L929 cells were examined under a light microscope (original magnification, 100×) at 24 h, 48 h, and 72 h. Data represent the mean ± SD; *n* = 6; * *p* < 0.05 (significant difference compared with the control group).

The hemocompatibility of HPCT on rabbit red blood cells was evaluated using different concentrations of HPCT aqueous solution. The results were shown in Figure 4A and Figure 4B. Neither the experimental concentrations of HPCT nor saline caused rupture of rat erythrocytes, whether the test was performed using whole blood or 2% erythrocytes. The hemolysis rate of test concentration of HPCT was less than 5%, which meets the requirements of the hemolysis rate of medical materials.

In addition, according to “ISO 10993-5-2016 Biological evaluation of medical devices”, the cytotoxicity and cytocompatibility of the HPCT hydrogel were evaluated by the effect on the growth of mouse fibroblast L929 [47].

Figure 4D showed the microscopic images of L929 cells under the influence of different concentrations of HPCT (The green background is due to the optical filter). The L929 cells grew adherently, dispersed evenly and had a clear outline. There was no significant change in cell morphology between different groups, such as cell shrinkage or cytoplasmic vacuolization. Moreover, MTT results (Figure 4C) revealed that the relative proliferation rate of cells in each group was more than 80% at three time points after HPCT treatment. The above data disclosed that HPCT was non-toxic to L929 cells at the concentrations tested.

#### 3.4.2. HPCT Hydrogel Has Satisfactory Histocompatibility and Degradability

After determining that the HPCT hydrogel had great cytocompatibility in vitro, we injected it into the subcutaneous tissue of the rat back to further determine its histocompatibility and degradability in vivo.

**Figure 5:**
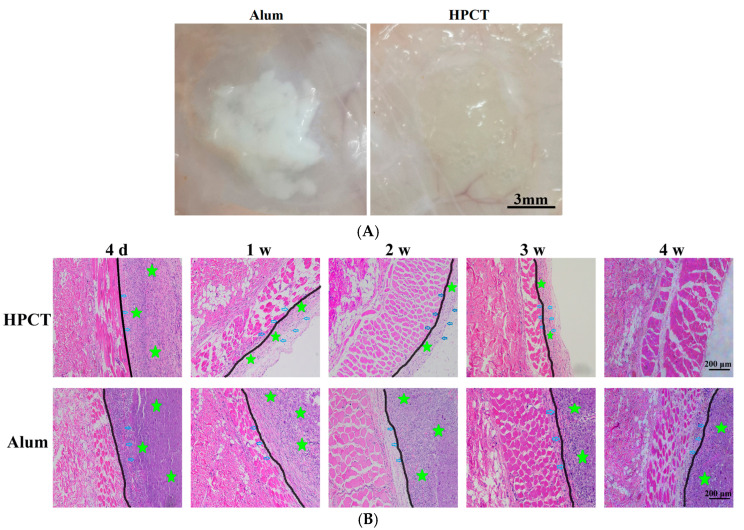
(**A**) The initial state of the alum and HPCT hydrogels after subcutaneous injection into rats. (**B**) H&E staining images at different times (the green pentagram areas are the subcutaneous residual material, the blue arrows show the approximate boundaries of residual material and tissue, and the black lines are the boundaries of the different areas).

Figure 5A is the state of HPCT and alum at 10 min after injection into rats. After subcutaneous injection of HPCT into rats, the gel was formed in a short period of time. H&E staining was performed after tissue sections containing materials were obtained, as shown in Figure 5B. After 4 days, there was a specific inflammatory response at the injection site for both materials. In the HPCT group, the inflammatory reaction had almost disappeared one week after the injection, the thickness of the material decreased, and the degradation was almost completed in the fourth week. The inflammatory response in the alum group persisted, with no significant reduction in inflammatory cells over the 4 weeks of experimental observation, and little absorption of alum injected subcutaneously. Throughout the degradation experiments, no adverse phenomena, such as tissue proliferation and damage, were observed at the injection sites of both groups of rats.

As an adjuvant for human and animal use, ensuring complete safety is paramount. Although aluminum adjuvants are not compatible with blood and have some negative effects on the body, the benefits outweigh the disadvantages and are, therefore, widely used. In our philosophy, an ideal adjuvant should be able to degrade gradually after it has exerted its optimal effect. Therefore, the adjuvant should be compatible with blood.

Taken together, based on the results of in vitro cytotoxicity evaluation and in vivo degradation in histocompatibility experiments, we proved that HPCT has excellent biocompatibility and can ensure its safety as an adjuvant for injection vaccines [48].

### 3.5. Enzymatic Hydrolysis and BSA Release of HPCT Hydrogel In Vitro

**Figure 6:**
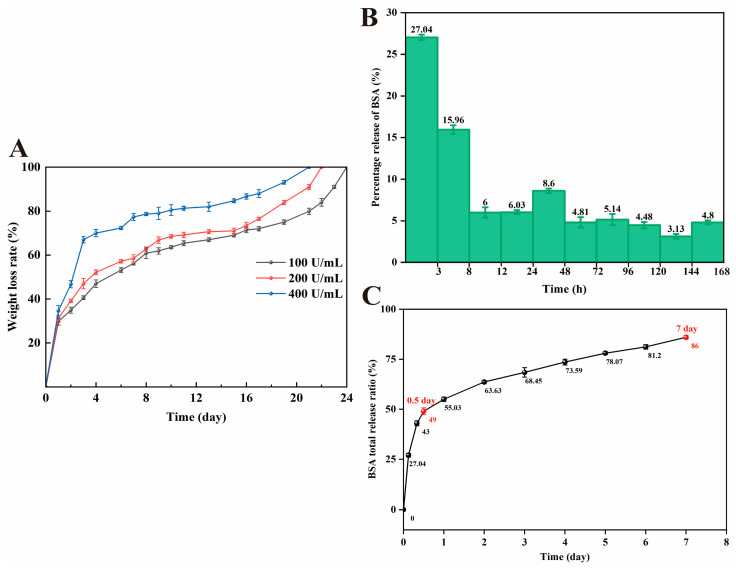
(**A**) The degradation curve of the HPCT hydrogel in different concentrations of lysozyme. (**B**) Percentage of BSA released in stages from the HPCT hydrogels (weight of BSA released per stage/total weight of BSA). (**C**) Total release rate for BSA from the HPCT hydrogel.

Lysozyme is a well-known enzyme found in many organisms and is often used as a model protein for structural, enzymatic, and immunological studies. Lysozyme can hydrolyze the β-(1→4)-glucosidic bond [49]. In addition, a study showed that chitosan is mainly depolymerized by lysozyme in human serum [50]. Studying the effect of lysozyme on the degradation of HPCT can allow for a preliminary understanding of its absorption in vivo. The degradation of HPCT hydrogel in different concentrations of lysozyme solution is shown in Figure 6A. Before the fourth day, the hydrogel degraded rapidly. After that, the degradation rate of the hydrogel tended to be gentle. It was completely degraded after 3 weeks in the 400 U/mL lysozyme system.

The high porosity and adhesion of hydrogels make them suitable for slow-release materials, such as carriers for protein release [51,52]. The sustained-release effect of a protein-loaded hydrogel depends on the mode of action of the protein and hydrogel, the ratio of protein size to hydrogel pore size, and other factors. The hydrogel carrier with excellent performance can reduce the frequency of administration and help the slow and stable release of protein and, thus, greatly improving the bioavailability of the protein [53].

The release of BSA loaded in HPCT hydrogel was tested in vitro (Figure 6B,C). The BSA in the hydrogel had an obvious burst release phenomenon in the early stage; 43% of the total weight was released in the first eight hours. At 12 h, nearly half of the BSA was released (49%). Subsequent to this time, the release of BSA showed a slow and sustained trend, and the release rate exceeded 80% on the sixth day. The combination of BSA’s surface active ingredients and HPCT enhances the overall structural stability. Therefore, HPCT can effectively slow down the release of BSA. The study of hydrogel degradation behavior and protein release behavior also provides a basis for the design of animal experiment cycle of hydrogel as an adjuvant.

### 3.6. Detection of Specific Antibody Expression Level

In the previous experiments, we successfully synthesized HPCT and dissolved it to obtain HPCT hydrogels with a reversible temperature phase transition. It has a porous structure and suitable rheological properties. The hemolysis experiment, cytotoxicity experiment, and in vivo implantation experiment proved that the HPCT hydrogel had good biocompatibility. In addition, enzymatic hydrolysis experiments and protein release experiments further determined its properties as a carrier. On the basis of the above, we combined the 0.8% HPCT hydrogel with Brucella protein antigens to form a vaccine, and we carried out mouse immunization experiments to evaluate the potency of the HPCT hydrogel as an adjuvant for the brucellosis vaccine. 

**Figure 7:**
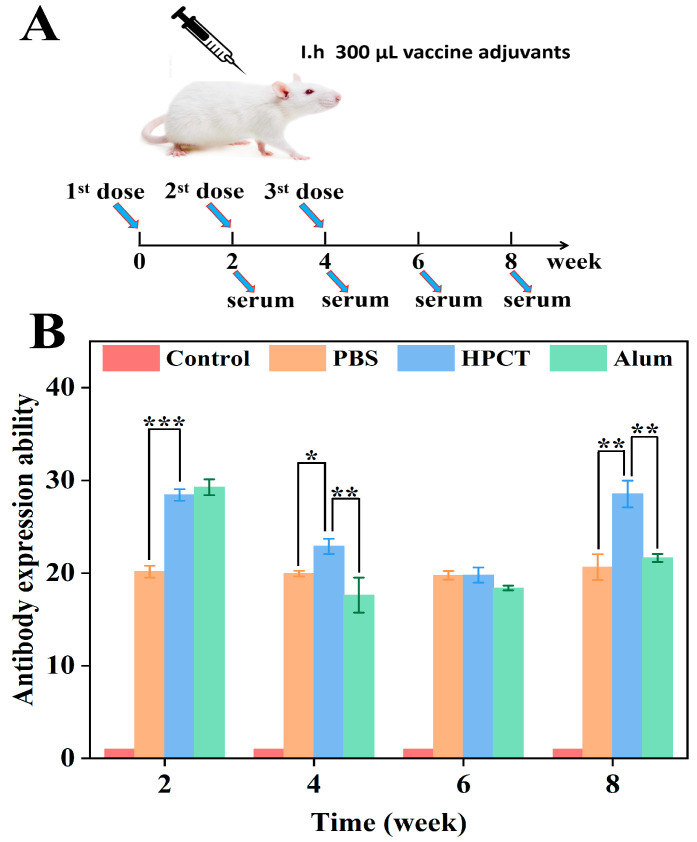
(**A**) Mouse immune program diagram. (**B**) Specific antibody expression levels in mouse serum at different time points. Data represent mean ± SD, *n* = 8, * *p* < 0.05, ** *p* < 0.01, and *** *p* < 0.001 (significant difference compared to other groups).

The immune process is shown in Figure 7A. The ELISA method is a designated diagnostic method for the detection of bovine brucellosis by the International Trade Organization, which has the advantages of being fast and accurate. Therefore, in this experiment, the brucella protein antigen was coated in a 96-well plate, and the specific antibody level in the serum of mice caused by each group of materials was evaluated by indirect ELISA [54].

The specific antibody levels in mice after immunization were shown in Figure 7B. At the second week after the first immunization, the alum adjuvant group produced the highest antibody level, followed by the HPCT hydrogel group, and there was no significant difference between the two groups (*p* > 0.05); both produced high levels of antibodies. At the 4th and 6th weeks, even though the antibody expression ability decreased. However, the antibody level in the HPCT hydrogel group was still higher than that in the alum group. At the 8th week, 4 weeks after the last booster immunization, the expression level of the HPCT hydrogel group was significantly higher than that of the alum group, and there was a significant difference between the two groups (*p* < 0.01). Combined with the previous protein release experiment of HPCT, it is speculated that the HPCT thermosensitive hydrogel has a sustained release effect on protein antigens and can form an antigen reservoir. The antigens are gradually released with the degradation of HPCT, while the alum group materials and protein antigens are simply mixed and do not form a dense structure such as covalent bonds. In addition, the blank control group did not contain the antigen protein, so there was a significant difference compared with other groups (*p* < 0.001). Therefore, the HPCT hydrogel material has the most significant enhancement in the specific expression of the Brucella protein antigen.

### 3.7. Detection of Related Cytokines 

Interferon-γ (IFN-γ) is a protein produced by T lymphocytes, natural killer cells and other immune cells. It is a marker cytokine produced by TH1 cells. IFN-γ plays an important role in immune regulation such as promoting antigen presentation and increasing macrophage lysosomal activity. Interleukin-4 (IL-4) is a pleiotropic cytokine that is considered a typical cytokine produced by TH2 cells. It is crucial for the development of T and B lymphocytes, as well as driving humoral immune responses and antibody production. In order to further study the effect of HPCT hydrogel on cellular immune response (Th1) and humoral immune response (Th2), the expression levels of IFN-γ (Th1 cytokine) and IL-4 (Th2 cytokine) were detected at various time periods. 

**Figure 8:**
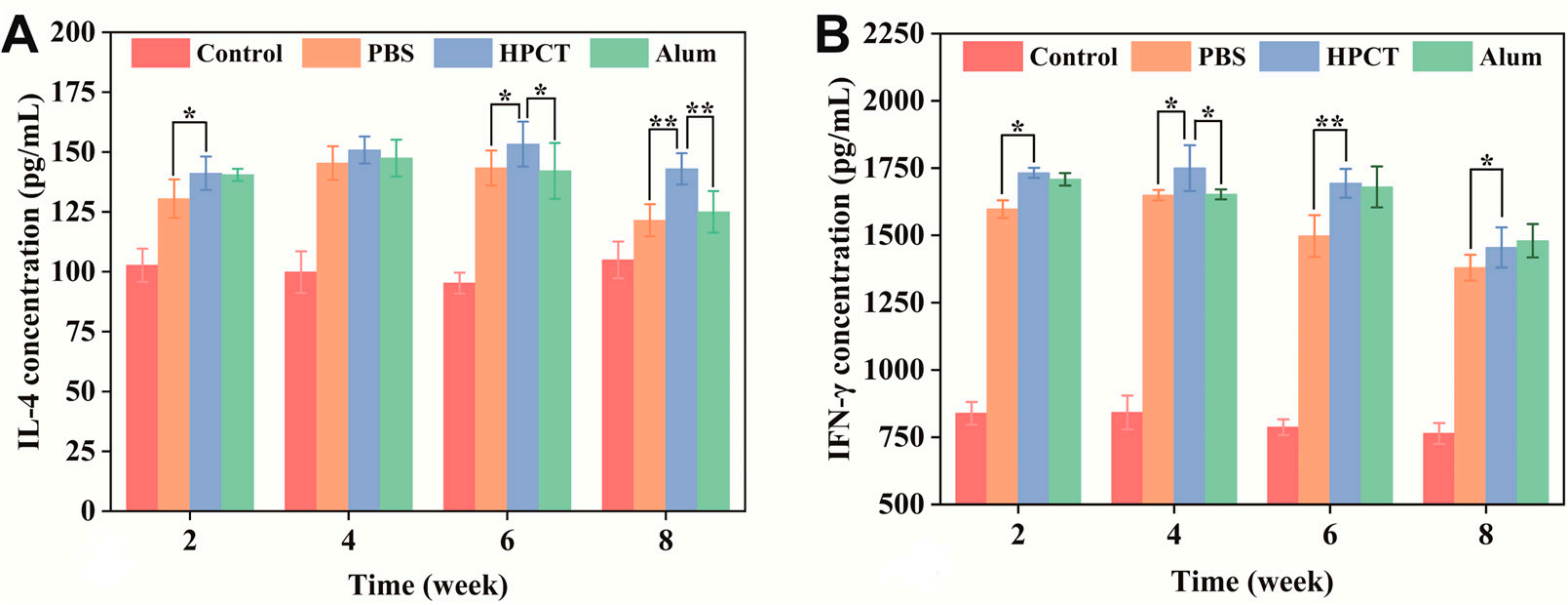
The expression levels of cytokines IL-4 (**A**) and IFN-γ (**B**) in serum at different times after immunization. Data represent mean ± SD, *n* = 8, * *p* < 0.05 and ** *p* < 0.01 (significant difference compared to other groups).

The results are shown in Figure 8A. There was no significant difference in IL-4 levels between HPCT hydrogel group and alum group at 2 and 4 weeks after the first immunization (*p* > 0.05). At 6 and 8 weeks, the expression of IL-4 in HPCT hydrogel group was significantly higher than that in alum group (*p* < 0.05) and other groups. It shows that HPCT hydrogel as an adjuvant can promote the body to produce more cytokines to trigger humoral immunity. The cellular immune response depends on the interaction between T lymphocytes and antigen presenting cells. As the most effective defense response to eliminate intracellular parasitic microorganisms, it is most difficult to be efficiently expressed. Figure 8B shows the expression level of the cellular immune factor IFN-γ. At the 4th week, the HPCT hydrogel group had the highest IFN-γ expression level, which was significantly different from the positive control alum group (*p* < 0.05). In the sixth and eighth weeks, the IFN-γ expression levels in the HPCT hydrogel group were not significantly different from those in the alum group, and both were higher than those in the PBS group. This indicates that the ability of HPCT hydrogel materials to induce the expression of cellular immune factor IFN-γ is comparable to that of commonly used aluminum hydroxide adjuvants. In addition, all experimental groups were significantly higher than the control group without the protein (*p* < 0.001). In summary, the use of an HPCT hydrogel as an adjuvant for the Brucella protein antigen can trigger effective immune response levels, in particular, it can significantly improve the level of specific antibody expression and humoral immune response in this experiment.

We believe that there are multiple reasons why HPCT hydrogels can promote immune responses. On the one hand, as mentioned earlier, chitin derivatives can increase B-cell activity in vivo, which in turn promotes humoral immunity; on the other hand, hydrogels with unique temperature-sensitive properties can bind antigens firmly and release them gradually with degradation. These two novel advantages are not found in general adjuvants (e.g., alum).

## 4. Conclusions

In a groundbreaking development, we introduced the temperature-sensitive HPCT hydrogel as an adjuvant in the brucellosis vaccine for the very first time. The stable three-dimensional structure of the HPCT hydrogel formed after gel formation at body temperature can slowly release the drug and prolong the drug release cycle and, thus, reduces the frequency of injection and acute drug exposure to tissues, improving the efficacy of the drug. These are excellent properties that are not found in traditional adjuvants. Subsequent assessments of specific immunity, humoral immunity, and cellular immune factors demonstrated that the HPCT hydrogel outperformed the commonly employed alum adjuvant. HPCT hydrogel, a natural polysaccharide material renowned for its exceptional biocompatibility and degradability, exhibits substantial promise as a vaccine adjuvant not only for brucellosis. We hypothesize that it shows good results in other antigen encapsulations as well. Consequently, the HPCT hydrogel holds immense potential as a vaccine adjuvant with a bright outlook for applications. Nonetheless, the binding mode and release kinetics between HPCT hydrogels and antigenic proteins need to be further explored and revealed in subsequent experiments. In addition, setting the optimal injection frequency and interval period of the hydrogel based on the release cycle of the drug and the degradation time of the hydrogel requires a large number of experiments to corroborate.

## Figures and Tables

**Table 1:** Analysis results of organic elements of chitin and HPCT.

Sample	N (%)	C (%)	H (%)	C/N Ratio
CT	6.66	45.27	5.27	6.79
HPCT	5.27	46.41	6.20	8.81

## Data Availability

The data that support the findings of this study are contained within the article and the Supplementary Materials.

## Supplementary Materials

The following supporting information can be downloaded at: https://www.mdpi.com/article/10.3390/polym16192815/s1, Step S1: Steps for detection of specific antibody expression by Indirect Enzyme-Linked Immunosorbent assay.

## References

[1] Nicoletti P. (2010). Brucellosis: Past, present and future. Prilozi.

[2] Shakir R. (2021). Brucellosis. J. Neurol. Sci..

[3] Ulu-Kilic A., Metan G., Alp E. (2013). Clinical presentations and diagnosis of brucellosis. Recent. Pat. Antiinfect. Drug Discov..

[4] de Figueiredo P., Ficht T.A., Rice-Ficht A., Rossetti C.A., Adams L.G. (2015). Pathogenesis and immunobiology of brucellosis: Review of Brucella-host interactions. Am. J. Pathol..

[5] Skalsky K., Yahav D., Bishara J., Pitlik S., Leibovici L., Paul M. (2008). Treatment of human brucellosis: Systematic review and meta-analysis of randomised controlled trials. BMJ.

[6] Perkins S.D., Smither S.J., Atkins H.S. (2010). Towards a Brucella vaccine for humans. FEMS Microbiol. Rev..

[7] Avila-Calderón E.D., Lopez-Merino A., Sriranganathan N., Boyle S.M., Contreras-Rodríguez A. (2013). A History of the Development of *Brucella* Vaccines. BioMed Res. Int..

[8] (2005). Guideline on Adjuvants in Vaccines for Human Use.

[9] Coffman R.L., Sher A., Seder R.A. (2010). Vaccine adjuvants: Putting innate immunity to work. Immunity.

[10] Firdaus F.Z., Skwarczynski M., Toth I., Thomas S. (2022). Developments in Vaccine Adjuvants. Vaccine Design: Methods and Protocols, Volume 3. Resources for Vaccine Development.

[11] Oleszycka E., Lavelle E.C. (2014). Immunomodulatory properties of the vaccine adjuvant alum. Curr. Opin. Immunol..

[12] Kool M., Fierens K., Lambrecht B.N. (2012). Alum adjuvant: Some of the tricks of the oldest adjuvant. J. Med. Microbiol..

[13] Clements C.J., Griffiths E. (2002). The global impact of vaccines containing aluminium adjuvants. Vaccine.

[14] Liu H., Liu J., Qi C., Fang Y., Zhang L., Zhuo R., Jiang X. (2016). Thermosensitive injectable in-situ forming carboxymethyl chitin hydrogel for three-dimensional cell culture. Acta Biomater..

[15] Wang D., Zou Y., Wang N., Wu J. (2022). Chitosan hydrochloride salt stabilized emulsion as vaccine adjuvant. Carbohydr. Polym..

[16] AbdelAllah N.H., Gaber Y., AbdelGhani S., Rashed M.E., Azmy A.F. (2021). Chitosan and alginate salt as biomaterials are potential natural adjuvants for killed cholera vaccine. J. Biomed. Mater. Res. A.

[17] Mallakpour S., Azadi E., Hussain C.M. (2021). Chitosan, alginate, hyaluronic acid, gums, and β-glucan as potent adjuvants and vaccine delivery systems for viral threats including SARS-CoV-2: A review. Int. J. Biol. Macromol..

[18] Pellis A., Guebitz G.M., Nyanhongo G.S. (2022). Chitosan: Sources, Processing and Modification Techniques. Gels.

[19] Suzuki K., Okawa Y., Hashimoto K., Suzuki S., Suzuki M. (1984). Protecting effect of chitin and chitosan on experimentally induced murine candidiasis. Microbiol. Immunol..

[20] Nishimura K., Nishimura S., Nishi N., Tokura S., Azuma I., Nishimura K., Nishimura S., Nishi N., Tokura S., Azuma I. (1986). Immunological Activity of Chitin Derivatives. Chitin in Nature and Technology.

[21] Ahmad S.I., Ahmad R., Khan M.S., Kant R., Shahid S., Gautam L., Hasan G.M., Hassan M.I. (2020). Chitin and its derivatives: Structural properties and biomedical applications. Int. J. Biol. Macromol..

[22] Li X., Min M., Du N., Gu Y., Hode T., Naylor M., Chen D., Nordquist R.E., Chen W.R. (2013). Chitin, chitosan, and glycated chitosan regulate immune responses: The novel adjuvants for cancer vaccine. Clin. Dev. Immunol..

[23] Sadeghi S., Bandehpour M., Haji Molla Hoseini M., Sharifnia Z. (2021). Intranasal administration of immunogenic poly-epitope from influenza H1N1 and H3N2 viruses adjuvanted with chitin and chitosan microparticles in BALB/c mice. Iran. J. Basic. Med. Sci..

[24] Zhao H., Wang H., Hu Y., Xu D., Yin C., Han Q., Zhang J. (2021). Chitosan Nanovaccines as Efficient Carrier Adjuvant System for IL-12 with Enhanced Protection Against HBV. Int. J. Nanomed..

[25] Yu S., Hao S., Sun B., Zhao D., Yan X., Jin Z., Zhao K. (2020). Quaternized Chitosan Nanoparticles in Vaccine Applications. Curr. Med. Chem..

[26] Xu Y., Xu Y., Bi B., Hou M., Yao L., Du Q., He A., Liu Y., Miao C., Liang X. (2020). A moldable thermosensitive hydroxypropyl chitin hydrogel for 3D cartilage regeneration in vitro and in vivo. Acta Biomater..

[27] Zhang Y.Q., Zhang K. (2014). Simultaneous determination of the degree of substitution and moisture content of hydroxypropyl chitosan by elemental analysis. Chin. J. Anal. Lab..

[28] Sæbø I., Bjørås M., Franzyk H., Helgesen E., Booth J. (2023). Optimization of the Hemolysis Assay for the Assessment of Cytotoxicity. Int. J. Mol. Sci..

[29] Tibor A., Decelle B., Letesson J.J. (1999). Outer Membrane Proteins Omp10, Omp16, and Omp19 of Brucella spp. Are Lipoproteins. Infect. Immun..

[30] Zhi F., Zhou D., Li J., Tian L., Zhang G., Jin Y., Wang A. (2020). Omp16, a conserved peptidoglycan-associated lipoprotein, is involved in Brucella virulence in vitro. J. Microbiol..

[31] Pasquevich K.A., Carabajal M.V., Guaimas F.F., Bruno L., Roset M.S., Coria L.M., Rey Serrantes D.A., Comerci D.J., Cassataro J. (2019). Omp19 Enables Brucella abortus to Evade the Antimicrobial Activity From Host’s Proteolytic Defense System. Front. Immunol..

[32] Vinh D.C., Abel L., Bastard P., Cheng M.P., Condino-Neto A., Gregersen P.K., Haerynck F., Cicalese M.P., Hagin D., Soler-Palacín P. (2021). Harnessing Type I IFN Immunity Against SARS-CoV-2 with Early Administration of IFN-β. J. Clin. Immunol..

[33] Paul W.E. (2015). History of interleukin-4. Cytokine.

[34] Feng F., Liu Y., Hu K. (2004). Influence of alkali-freezing treatment on the solid state structure of chitin. Carbohydr. Res..

[35] Yamaguchi Y., Nge T.T., Takemura A., Hori N., Ono H. (2005). Characterization of Uniaxially Aligned Chitin Film by 2D FT-IR Spectroscopy. Biomacromolecules.

[36] Yuan M., Bi B., Huang J., Zhuo R., Jiang X. (2018). Thermosensitive and photocrosslinkable hydroxypropyl chitin-based hydrogels for biomedical applications. Carbohydr. Polym..

[37] Wang P., Lv X., Zhang B., Wang T., Wang C., Sun J., Zhang K., Wu Y.n., Zhao J., Zhang Y. (2021). Simultaneous determination of molar degree of substitution and its distribution fraction, degree of acetylation in hydroxypropyl chitosan by 1H NMR spectroscopy. Carbohydr. Polym..

[38] Peng Y., Han B., Liu W., Xu X. (2005). Preparation and antimicrobial activity of hydroxypropyl chitosan. Carbohydr. Res..

[39] Jones C.D., Lyon L.A. (2003). Shell-Restricted Swelling and Core Compression in Poly(N-isopropylacrylamide) CoreShell Microgels. Macromolecules.

[40] Doberenz F., Zeng K., Willems C., Zhang K., Groth T. (2020). Thermoresponsive polymers and their biomedical application in tissue engineering—A review. J. Mater. Chem. B.

[41] Wu Y., Wenger A., Golzar H., Tang X. (2020). 3D bioprinting of bicellular liver lobule-mimetic structures via microextrusion of cellulose nanocrystal-incorporated shear-thinning bioink. Sci. Rep..

[42] Horváth S. (1980). Cytotoxicity of drugs and diverse chemical agents to cell cultures. Toxicology.

[43] Blac J. (1984). Systemic effects of biomaterials. Biomaterials.

[44] Tyan Y.-C., Yang M.-H., Chang C.-C., Chung T.-W., Chun H.J., Reis R.L., Motta A., Khang G. (2020). Biocompatibility of Materials for Biomedical Engineering. Biomimicked Biomaterials: Advances in Tissue Engineering and Regenerative Medicine.

[45] Jaffer I.H., Weitz J.I. (2019). The blood compatibility challenge. Part 1: Blood-contacting medical devices: The scope of the problem. Acta Biomater..

[46] Maitz M.F., Martins M.C.L., Grabow N., Matschegewski C., Huang N., Chaikof E.L., Barbosa M.A., Werner C., Sperling C. (2019). The blood compatibility challenge. Part 4: Surface modification for hemocompatible materials: Passive and active approaches to guide blood-material interactions. Acta Biomater..

[47] Hou L., Sun X. (2017). Biological Evaluation of Medical Devices—Part 5:Tests for In Vitro Cytotoxicity. Inspection and Quarantine of the People’s Republic of China. https://kns.cnki.net/kcms2/article/abstract?v=cF0fONyw0YJxIX1tlEtAQZwQyyc2s6iiKFwYtGtplV7GG0x7j_fN-cWHDu2wcdKTFvb9-NJ2bLG-Q9sYDkJtzEIwIxP3GqgOX5Qd5sAfFxDoxbCDU0LZbcuamssUuKvL5hk24dlbI5BbPFp9usA1JIOHrAyUi_OnPsBaN6AECIkU12xG4ExB1V72ojGAbAmn&uniplatform=NZKPT&language=CHS.

[48] Raut H.K., Das R., Liu Z., Liu X., Ramakrishna S. (2020). Biocompatibility of Biomaterials for Tissue Regeneration or Replacement. Biotechnol. J..

[49] Mukhametova L.I., Zherdev D.O., Kuznetsov A.N., Yudina O.N., Tsvetkov Y.E., Eremin S.A., Krylov V.B., Nifantiev N.E. (2024). Fluorescence-Polarization-Based Assaying of Lysozyme with Chitooligosaccharide Tracers. Biomolecules.

[50] Vårum K.M., Myhr M.M., Hjerde R.J., Smidsrød O. (1997). In Vitro degradation rates of partially n-acetylated chitosans in human serum. Carbohydr. Res..

[51] Ruel-Gariépy E., Chenite A., Chaput C., Guirguis S., Leroux J.C. (2000). Characterization of thermosensitive chitosan gels for the sustained delivery of drugs. Int. J. Pharm..

[52] Kim G.O., Kim N., Kim D.Y., Kwon J.S., Min B.H. (2012). An electrostatically crosslinked chitosan hydrogel as a drug carrier. Molecules.

[53] Bajpai A.K., Bajpai J., Saini R., Gupta R. (2011). Responsive Polymers in Biology and Technology. Polym. Rev..

[54] Lin A.V., Hnasko R. (2015). Indirect ELISA. ELISA: Methods and Protocols.

