# Supplementary material for: A Thermosensitive and Degradable Chitin-Based Hydrogel as a Brucellosis Vaccine Adjuvant
Source: Polymers (Basel). 2024 Oct 4;16(19):2815. doi: 10.3390/polym16192815 (PMC11478596; doi:10.3390/polym16192815)
Supplement: Supplementary file 1 [file polymers-16-02815-s001.zip › polymers-3198300-supplementary.pdf]

A Thermosensitive and Degradable Chitin-Based Hydrogel as a Brucellosis Vaccine Adjuvant

Ruibao Ju [1], Yanjing Lu [2], Zhiwen Jiang [1], Jinhua Chi [1], Shuo Wang [1], Wanshun Liu [1], Yanbo Yin [2,*] and Baoqin Han [1,*]

[1]Laboratory of Biochemistry and Biomedical Materials, College of Marine Life Sciences, Ocean University of China, Qingdao 266003, China

[2]College of Veterinary Medicine, Qingdao Agricultural University, Qingdao 266109, China

*Correspondence address. E-mail: baoqinh@ouc.edu.cn, yanboyin2011@163.com

Step S1. Steps for detection of specific antibody expression by Indirect Enzyme-Linked Immunosorbent assay:

(1) Encapsulation: Dilute recombinant Brucella protein antigen to 1.5 μg/mL with 0.1 mol/L $NaHCO_3$, add it to the 96-well plate in the amount of 100 μL per well, and incubate at 4°C for more than 12 h. The sample was then incubated for 12 h at 4°C.

(2) Closure: Discard the coating solution in the 96-well plate, wash the plate with PBST for three times, shake out the liquid in the wells, add 200 μL of skimmed milk powder closure solution to each well, and incubate for 2 h at 37°C.

(3) Add primary antibody: discard the liquid in the plate, wash the plate with PBST three times, dry the liquid in the wells, add 200 μL of different groups of mouse serum diluted 150-fold with skimmed milk powder into each well, and incubate at 37°C for 2 hours.

(4) Add secondary antibody: discard the liquid in the wells, wash the plate with PBST three times, shake off the liquid in the wells, add 100 μL of goat anti-mouse IgG secondary antibody diluted with skimmed milk powder to each well, and leave it for 1 h at room temperature.

(5) Color development: Discard the liquid in the wells, wash the plate with PBST for five times, shake off the liquid in the wells, add 50 μL of TMB color development solution into each well, incubate for 20 min under low light conditions. The reaction was terminated by adding 50 μL of 2 mol/L H_2SO_4 to each well, and the OD value at 450 nm was measured by an enzyme marker within 15 min. Antibody expression ability was expressed as the ratio of the OD value of each group to the OD value of the control group.
